# 2419. Increasing Misattribution of Bacteremia as CLABSIs by NHSN Definitions: A Tertiary Care Center Perspective

**DOI:** 10.1093/ofid/ofad500.2039

**Published:** 2023-11-27

**Authors:** Misti G Ellsworth, Valerie Ausborn, Bela Patel, Phillip Chang, Luis Ostrosky-Zeichner

**Affiliations:** UTHealth, Houston, Texas; Memorial Hermann Health System, Houston, Texas; University of Texas Health Science Center, Houston, Texas; Memorial Hermann Health System, Houston, Texas; McGovern Medical School. UTHealth, Texas, Texas

## Abstract

**Background:**

The US has experienced a significant decrease in CLABSI rates over time but is stalling. NHSN has rigid definitions for secondary BSI attribution. We reviewed CLABSIs in a large tertiary acute care hospital to determine the frequency and trends of potential misattribution.

**Methods:**

We reviewed 282 CLABSIs cases from 2019 to 2023 at a large tertiary hospital. A team of infection preventionists with high inter-rate reliability adjudicated each case strictly following NHSN methodology. A clinical review was performed by two hospital ID-trained epidemiologists to explore misattributed with an alternative primary infection.

**Results:**

Of 282 CLABSI cases, 65% occurred in adults and 35% in children. 122 cases (43%) had a potential primary infection. The potential primary infections included: 33 (27%) pneumonia, 28 (23%) gastrointestinal infections, 6 (5%) urinary infections, 4 (3%) surgical infections, 13 (11%) skin and soft tissue infections, 11 (9%) vascular infections, 5 (4%) bacterial translocation, 8 (6.5%) infections that were present on admission, 4 (3%) CNS infections, 2 (1.6%) NEC infections, 2 (1.6%) musculoskeletal, 2 (1.6%) contaminates and 4 (3%) other non-NHSN specified sites. Pneumonia was the most frequent misattribution failing to meet imaging, laboratory, or documentation requirements within the infection window period (IWP). Intra-abdominal infection was the second most frequent cause of misattribution due to not meeting the imaging or matching site culture within the IWP. For the other categories, the most frequent reasons for not meeting the primary criteria included lack of matching culture, lack of clinical symptoms (often in intubated patients), or in the endocarditis category, the complexity of the criteria even with positive blood cultures and presence of vegetations. The graph shows an increasing proportion of misclassification over time.

Percent of Misattributed CLABSI by Fiscal Year

**Figure:**
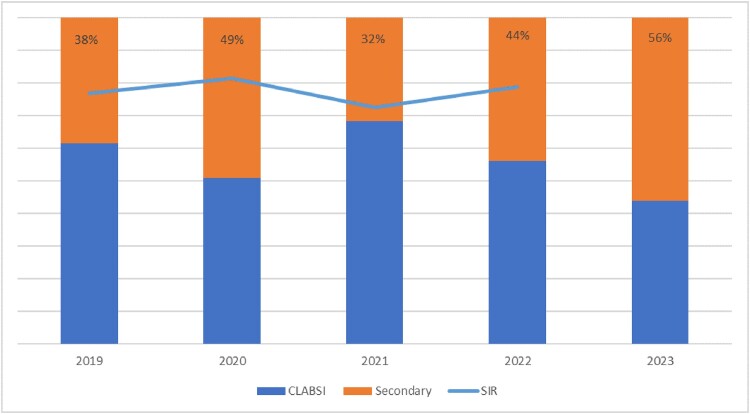

**Conclusion:**

NHSN definitions and rules are becoming too prescriptive and may no longer be applicable to the complexity of cases, overestimating CLABSIs. As hospitals commit to CLABSI reduction, accurate and specific definitions are key in focusing efforts and attention and avoiding unfair penalties.

**Disclosures:**

**Luis Ostrosky-Zeichner, MD, FACP, FIDSA, FSHEA, FECMM, CMQ**, Astellas: Grant/Research Support|Cidara: Advisor/Consultant|Cidara: Advisor/Consultant|F2G: Advisor/Consultant|Gilead: Advisor/Consultant|Gilead: Grant/Research Support|GSK: Advisor/Consultant|Melinta: Advisor/Consultant|NIH: Grant/Research Support|Pfizer: Advisor/Consultant|Pfizer: Grant/Research Support|Pfizer: Honoraria|Pulmocide: Grant/Research Support|Scynexis: Grant/Research Support|T2 Biosystems: Grant/Research Support|Viracor: Advisor/Consultant

